# Neurotrophic, Cytoprotective, and Anti-inflammatory Effects of St. John's Wort Extract on Differentiated Mouse Hippocampal HT-22 Neurons

**DOI:** 10.3389/fphar.2017.00955

**Published:** 2018-01-18

**Authors:** Gabriel A. Bonaterra, Anna Schwendler, Julian Hüther, Hans Schwarzbach, Anja Schwarz, Christiane Kolb, Heba Abdel-Aziz, Ralf Kinscherf

**Affiliations:** ^1^Department of Medical Cell Biology, Institute for Anatomy and Cell Biology, Philipps-University of Marburg, Marburg, Germany; ^2^Steigerwald Arzneimittelwerk GmbH, Scientific Department, Darmstadt, Germany

**Keywords:** anti-inflammatory, differentiated hippocampal neurons, human macrophages, neuronal plasticity, NMDA/glutamate excitotoxicity, St. John's wort, STW3-VI, THP-1

## Abstract

**Introduction:** Since ancient times *Hypericum perforatum* L. named St. John's wort (SJW), has been used in the management of a wide range of applications, including nervous disorders. Development of mood disorders are due to alterations in glutamate metabolism, initiation of inflammatory pathways, and changes of the neuronal plasticity. Previous studies suggest that the glutamatergic system contributes to the pathophysiology of depression. Extracts of SJW have been recommended for the treatment of depression. The aim of the present *in vitro* study was to evaluate the action of STW3-VI, a special SJW extract in differentiated mouse hippocampal HT-22 neurons. We evaluated the stimulation of neurogenesis, the protective effect against glutamate or N-methyl-D-aspartate receptor induced-excitotoxicity and its anti-inflammatory properties in LPS-activated human macrophages.

**Results:** After 48 h treatment, STW3-VI stimulated the neurite formation by 25% in comparison with the control and showed protective effects against glutamate- or NMDA-induced cytotoxicity by significantly increasing the viability about +25 or +50%. In conjunction with these effects, after pretreatment with STW3-VI, the intracellular reduced glutathione content was significantly 2.3-fold increased compared with the neurons incubated with glutamate alone. Additionally, pre-treatment of human macrophages with STW3-VI showed anti-inflammatory effects after 24 or 48 h concerning inhibition of LPS-induced TNF release by −47.3 and −53.8% (24 h) or −25.0 to −64.8% (48 h).

**Conclusions:** Our data provide new evidence that STW3-VI protects hippocampal cells from NMDA- or glutamate-induced cytotoxicity. Moreover, our results indicate a morphological remodeling by increasing neurite outgrowth and activation of the anti-inflammatory defense by inhibition of the cytokine production in human macrophages after STW3-VI treatment. These protective, neurotrophic and anti-inflammatory properties may be beneficial in the treatment of depressive disorders.

## Introduction

Neurological injury and chronic stress are important factors in the pathophysiology of depression (Lazaratou et al., 2010). Recurrent stress to the cerebral cortex activates the hypothalamic-pituitary-adrenal (HPA) axis (Sartori et al., 2012), which causes HPA axis hyperfunction, increases adrenal cortical hormone levels, and induces hippocampal neuronal injury (Pariante, 2009). Stress and neurological injury are naturally diverse; however, there are common mechanisms of cellular damage. In this context, inflammation and toxicity of glutamate play a major role in a variety of neurological disorders like anxiety, Alzheimer's or Parkinson's disease, depression, epilepsy, and ischemic stroke (Tokarski et al., 2008; Haroon et al., 2016). Additionally, innumerous evidence exists that adaptation to chronic stress involves response from the neuroendocrine, but from the immune system as well (Dinan, 2009). Major depression is frequently induced by psychosocial stress and is associated with modifications in the immune system as well as in the HPA axis (Dinan, 2009). Depression has been linked to a pro-inflammatory response, because cytokines like interleukin 6 (IL-6) and tumor necrosis factor alpha (TNF) are increased (Miller et al., 2009; Grundmann et al., 2010). Pro-inflammatory cytokines have a positive and a negative effect on neuronal differentiation and proliferation and may be involved in molecular and cellular mechanisms associated with detrimental alterations in brain-immune communication fundamental for the development of neuropsychiatric syndromes

Glutamate, as an excitatory neurotransmitter in the mammalian central nervous system, can accumulate to an abnormally high concentration in the brain, which could initiate several neurodegenerative diseases (Sun et al., 2010; Hu et al., 2012). Moreover, a growing body of evidence exists, that the glutamatergic system, especially glutamate and N-methyl-D-aspartate (NMDA) receptors, leads to the physiopathology of depressive disorders (Serafini et al., 2013; Dantzer and Walker, 2014; Haroon et al., 2016). Furthermore, a special glutamate-mediated cell death pathway has been identified including hyperactivation of receptors (Molina-Hernández et al., 2008; Hu et al., 2012). Furthermore, it has been suggested that the combination of antidepressant medication which elevate norepinephrine levels and modify the glutamate dependent system could be an option in treating depression (Stoll et al., 2007; Molina-Hernández et al., 2008). The term “neural plasticity” also includes a large number of mechanisms, e.g., migration, proliferation, integration of new neurons, neurite growth, synaptogenesis, as well as the formation of mature synapses (Wainwright and Galea, 2013). Additionally, an association between depression, neuronal plasticity and hippocampal activity has recently been described (Wainwright and Galea, 2013). However, little is known about the effects of STW3-VI on the hippocampal plasticity and the possible association with cognitive impairment or depression (Pennington et al., 2009; Trofimiuk et al., 2011). In this context, it is tempting to speculate that the combined antioxidant and anti-inflammatory properties of STW3-VI extract leads to the antidepressant effects via normalization of an overactive HPA axis and may have an effect on hippocampal plasticity (Mennini and Gobbi, 2004; Grundmann et al., 2010; Huang et al., 2011). STW3-VI extract, is a clearly defined hypericum dry extract, which fulfills the requirements of the European Pharmacopeia (European Pharmacopoeia, 2008) and which is commercially available as marketed medicinal product, e.g., in Germany (Laif®, Steigerwald Arzneimittelwerk GmbH, Darmstadt, Germany) with clinically shown efficacy for moderate depressive episodes (Uebelhack et al., 2004; Gastpar et al., 2006). STW3-VI is frequently used to treat mild to moderate depression, with very few side effects (Mennini and Gobbi, 2004; Gastpar et al., 2006). The antidepressant action of STW3-VI seems to be partly mediated by the link between the immune-/neuroendocrine system and oxidative defense. In previous studies, undifferentiated immortalized HT-22 cells have been used as a hippocampal neuronal cell model; however, this cell line does not express NMDA receptors, thus it is resistant to excitotoxicity (Murphy et al., 1989; Davis and Maher, 1994). Nevertheless, mouse hippocampal neuronal HT-22 cells express NMDA receptors after differentiation (He et al., 2013). In undifferentiated mouse hippocampal neuronal HT-22 cells, a protective effect of STW3-VI against glutamate-induced cytotoxicity was described (Breyer et al., 2007). However, data concerning the effect of STW3-VI on differentiated mouse hippocampal neuronal HT-22 cells are missing until now.

Thus, the aim of our *in vitro* investigations was to determine whether STW3-VI improves neuronal plasticity and whether STW3-VI protects (i) differentiated mouse hippocampal neuronal HT-22 cells from the cytotoxic effects of glutamate, NMDA, or (ii) macrophages against the pro-inflammatory effects of LPS, associated with neurodegenerative diseases and depression.

## Materials and methods

### Extract

Steigerwald Arzneimittelwerk GmbH (Darmstadt, Germany) provided the SJW extract STW3-VI; the methods of extraction and characterization were described in previous publications (Breyer et al., 2007; Grundmann et al., 2010; Jungke et al., 2011). Briefly, the phytochemical composition of the St. John's Wort dried extract contained 0.10–0.3% total hypericins (as hypericin, naphthodianthrone), maximum 6.0% hyperforin (Phloroglucinderivat), and minimum 6.0% flavonoids (as rutin) (European Pharmacopoeia, 2008). The drug-extract ratio (DER) was 3–6:1 and the extractant was 80 Vol.-% Ethanol.

### Cell cultures

The immortalized mouse hippocampal neuronal HT-22 cells were cultured in Dulbecco's modified Eagle's medium (Capricorn Scientific GmbH, Ebsdorfergrund, Germany) supplied with 10% fetal bovine serum (FBS), 100 U/ml penicillin, 0.1 mg/ml streptomycin (PAA GmbH). 2.5 × 10^3^ HT-22 cells were seeded in 96-well plates (BD Falcon™ Becton Dickinson GmbH, Heidelberg, Germany), and after 24 h culture another 24 h differentiation as described afterwards, following 48 h pre-treatment with or without 0.1–1.0 μg/ml STW3-VI or 0.1 μM (0.03 μg/ml), −1.0 μM (0.3 μg/ml) desipramine (Sigma-Aldrich, Saint Louis, USA). Differentiation was performed in modified, serum-free medium (Dulbecco's modified Eagle's medium, 1x N2 supplement [Life Technologies AG, Carlsbad, USA]), 50 ng/ml nerve growth factor-β (NGF-β), 100 μM phorbol 12,13-dibutyrate (PDBu; Santa Cruz Biotechnology Inc., Dallas, USA), 100 μM dibutyryl cAMP (Santa Cruz Biotechnology Inc.), 100 U/ml penicillin, as well as 0.1 mg/ml streptomycin (PAA GmbH, Cölbe, Germany) (Suo et al., 2003). All treatments were performed in pre-warmed (37°C) differentiation medium with reduced NGF-β content (5 ng/ml) or with buffered Hank's solution (Capricorn Scientific GmbH, 5.26 mM KCl, 0.43 mM KH_2_PO_4_, 134.2 mM NaCl, 4.09 mM NaHCO_3_, 0.33 mM Na_2_HPO_4_, 5.44 mM glucose, 2 mM CaCl_2_, and 20 mM HEPES, pH 7.4; Chiricozzi et al., 2010). The human acute monocytic leukemia cell line THP-1 (DSMZ GmbH, Braunschweig, Germany) was cultured in RPMI 1640 (PAA GmbH); with 10% FBS (PAA GmbH); 100 U/ml penicillin, as well as 0.1 mg/ml streptomycin (PAA GmbH). 3.0 × 10^4^ THP-1 cells were seeded in 96-well plates (BD Falcon™). After 24 h differentiation with 160 nM phorbol-12-myristate-13-acetate (PMA, Sigma-Aldrich, St. Louis, MO, USA), the medium was changed, and the macrophages were pre-treated during 24 or 48 h with different concentrations of STW3-VI (40–100 μg/ml) and afterwards 3 h with or without 0.1 or 0.01 μg/ml LPS-EB from *E. coli* O111:B4 (Cayla-InvivoGen Europe, Toulouse, France). The antagonist LPS from *Rhodobacter sphaeroides* (LPS-RS, Cayla-InvivoGen) was used as control of the TLR4 specific binding by competitive inhibition at 100-fold excess of the agonist LPS-EB.

### NMDA receptor immunocytochemistry

After 48 h, differentiated mouse hippocampal neuronal HT-22 cells were immediately fixed (10 min) with 4% v/v paraformaldehyde (PFA/phosphate-buffered saline [PBS], pH 7.4), and blocked with 1% rabbit serum (PAA Laboratories GmbH, Pasching, Austria) in PBS. Thereafter the cells were incubated overnight at 5–7°C with goat anti murine NMDAζ1, C-20 (Santa Cruz Biotechnology Inc.), afterwards visualization was performed with anti-goat Cy3 conjugated antibody (Dianova Vertriebs-GmbH, Hamburg, Deutschland) and nuclei were counterstained with DAPI (Thermo Fisher Scientific, St. Leon-Rot, Germany). Photos of neurons were taken using a Zeiss Axiovert 135 microscope and processed with the software AxioVision Release 4.8.2 (Carl Zeiss GmbH, Göttingen, Germany).

### Neuronal plasticity test

2.5 × 10^3^ HT-22 cells were seeded in 96-well plates (BD Falcon™) and differentiated as described. Previously the viability at different concentrations of STW3-VI (0.1, 0.5, and 1.0 μg/ml) or desipramine [0.1 μM (0.03 μg/ml), 0.5 μM (0.15 μg/ml), and 1.0 μM (0.3 μg/ml)] was determined as described below. After treatment with 0.5 μg/ml of STW3-VI or 1.0 μM desipramine hydrochloride (Sigma-Aldrich) the differentiation and neurites growth was followed and photographs were taken at 0, 24, and 48 h, using an inverted microscope (Zeiss Axiovert 135) equipped with a X–Y motorized stage and AxioVision 4 Modul Mark & Find 2 (Carl Zeiss GmbH). The total number of neurites per cell—as described by others (Xu et al., 2011)—was determined by counting the number of neurites directly stemming from the soma using Image J 1.46r software (National Institutes of Health, Bethesda, USA).

### Viability assay

Cell viability was measured using PrestoBlue™ reagent (Invitrogen-Life Technologies GmbH, Darmstadt, Germany), according to manufacturer's protocol. PrestoBlue™ was directly added to the cells in the culture medium at a final concentration of 10%. Thereafter the optical density (OD) was measured at 570 and 600 nm (as reference) using a Sunrise ELISA-reader (Tecan, Salzburg, Austria). Results were expressed in % of cytotoxicity [100 − (OD_570/600nm_ of samples × 100/OD_570/600nm_ of control without substances)]. Afterwards, the cells were stained with crystal violet solution (0.04% crystal violet in 4% [v/v]) (Merck KGaA, Darmstadt, Germany), solubilized with 1% SDS/PBS and the absorbance was measured at 595 and 660 nm (as reference). The PrestoBlue™ absorbance was normalized against crystal violet.

### Biochemical determination of intracellular glutathione

The 25 × 10^4^ HT-22 cells were differentiated as described above and after differentiation were treated 24 h with STW3-VI and afterwards 6 h with glutamate or NMDA together with glycine (5 mM). The cells were washed three times with PBS, mixed with 2.5% sulfosalicylic acid (SSA), sonicated, and centrifuged. Thereafter, the clear supernatants were used to determine total glutathione (tGSH), oxidized glutathione/glutathione disulfide (GSSG), and reduced glutathione (red. GSH) according to the method of Tietze (1969), as described previously (Kinscherf et al., 1998). Red. GSH was calculated by subtraction of GSSG from tGSH. The protein concentration was determined spectrophotometrically using the Pierce BCA (bicinchoninic acid) Protein Assay (Thermo Scientific, Rockford, USA). The values of tGSH and GSSG were normalized against protein concentration.

### Human (h) TNF enzyme-linked immuno sorbent assay (ELISA)

The release of hTNF was measured using enzyme-linked immuno sorbent assay (ELISA). Therefore, 3 × 10^4^ THP-1 cells were seeded (100 μl medium/well) in 96-well plates (BD Falcon™) and were differentiated into macrophages using 0.1 μg/ml phorbol 12-myristate 13-acetate (PMA; Sigma-Aldrich, St. Louis, USA) in RPMI 1640 medium for 5 days. Afterwards, the macrophages were pre-treated with STW3-VI (24 or 48 h) and post-treated with 0.01 μg/ml ultrapure lipopolysaccharide (LPS-EB) from E. coli O111:B4 (Cayla—InvivoGen Europe, Toulouse France) for 3 h, that is exclusively recognized by toll-like receptor 4 (TLR4). The LPS antagonist from *R. sphaeroides* (LPS-RS) was used as control for specific binding to TLR4 by competitive inhibition at 100-fold excess of the agonist LPS-EB. After LPS treatment the culture medium was harvested, centrifuged at 500 × g (5 min) and the supernatant was stored at −20°C. Human TNF was determined in the culture medium using the assay DuoSet ELISA Development kit (R&D Systems Europe, Ltd., Abingdon, UK) according to the manufacturer's instructions. Microplates NUNC MaxiSorp™ (Thermo Fisher Scientific) 96 wells were used. The peroxidase reaction was visualized with 50 μl peroxidase substrate Sigma Fast™ o-phenylenediamine dihydrochloride (OPD) (Sigma-Aldrich, St. Louis, USA) (30 min; room temperature [RT]), and stopped with 25 μl 3 N HCl. The absorbance was measured at 490 nm, reference 655 nm. The adherent cells were fixed with 4% PFA-PBS and afterwards stained with crystal violet solution. The amount of hTNF released into the medium was normalized against crystal violet (0.04% crystal violet in 4% [v/v]). The results are expressed as percentage of the released hTNF after stimulation with LPS-EB, which was considered as 100%.

### Real time quantitative reverse transcription polymerase chain reaction (qRT-PCR)

RNA was extracted using peq-GOLD Isolation Systems TriFast™ (PEQLAB Biotechnologie GmbH, Erlangen, Germany) according to the manufacturer's instructions. RNA concentration was measured using a NanoDrop 2000c Spectrophotometer (Thermo Scientific, Schwerte, Germany). RNA integrity was analyzed using the RNA 6000 NanoChip kit and an Agilent 2100 Bioanalyzer (Agilent Technologies GmbH., Waldbronn, Germany). An aliquot of 0.5 μg total RNA was treated with 1 unit of RNAse (Thermo Fisher Scientific) (30 min; 37°C). Reverse transcription was performed with 500 ng oligo (dT)_12–18_ primer, 20 unit of the Affinity Script multiple temperature cDNA synthesis (Agilent Technologies) and 24 units of Ribo Lock™ RNAse inhibitor (Thermo Fisher Scientific) (1 h; 42°C). The cDNA was used for qRT-PCR using the QuantiTect primer assays (QIAGEN GmbH, Hilden, Germany). Data analyses and qPCR were performed using the Mx3005P™ QPCR System (Stratagene) and the relative standard curve method. The standard curve was generated from a pool of cDNA. For each sample, the relative amount was calculated by linear regression analysis from their respective standard curves. Amplified product was confirmed by melting point curve analysis (55–95°C).The mean expression of human ACTB (QT0009531, 146 bp) and RPLPO (QT01839887, 170 bp) or murine ACTB (QT01136772, 77 bp) and RPLPO (QT00249375, 125 bp) sequences were used as reference genes (housekeeping genes) to normalize the mRNA input. The mRNA expressions of human IL-6 (QT00083720, 107 bp), TNF-α (QT01079561, 104 bp), or murine IL-6 (QT00098875, 128 bp), TNF-α (QT00104006, 112 bp) were analyzed.

### Statistical methods

The SigmaPlot®-12 software (Systat Software GmbH, Erkrath, Germany) was used to carry out statistical analyses by the unpaired Student's *t-*test or Mann-Whitney *U-*test. Data are shown as mean + SEM. *p* ≤ 0.05 was considered as statistically significant.

## Results

### Differentiation induces morphological changes and expression of the NMDA receptor in mouse hippocampal neuronal HT-22 cells

**Figure 1:**
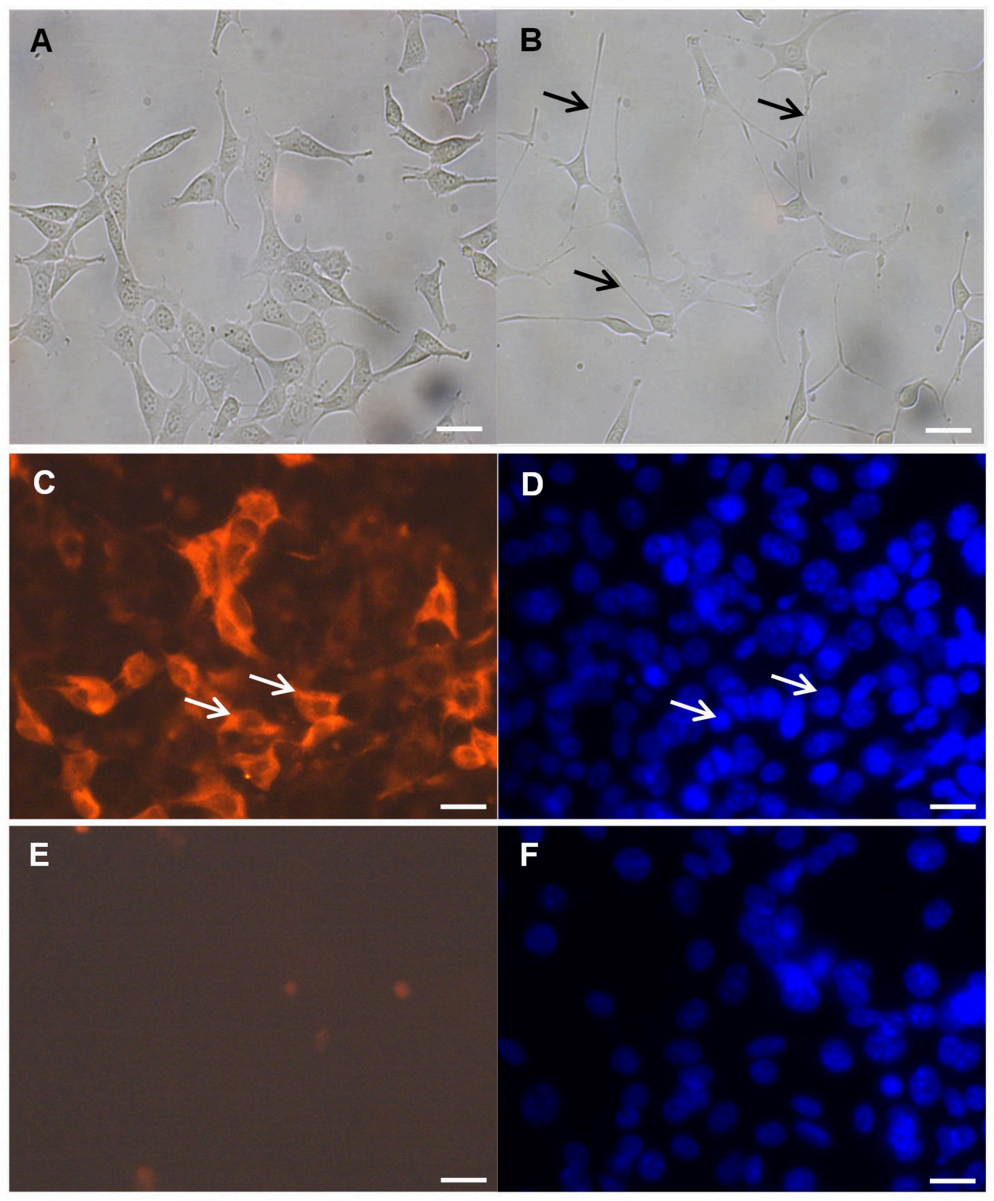
NMDA receptor immunocytochemistry. **(A)** Undifferentiated mouse hippocampal neuronal HT-22 cells and **(B)** after differentiation, black arrow indicate neurites. Differentiation conditions induce the expression of the NMDA receptor in the mouse hippocampal neuronal HT-22 cells after 48 h. **(C)** Representative images of the mouse hippocampal neurons labeled with anti-NMDA receptor antibody and **(D)** DAPI counterstain; white arrows indicate positive reaction **(C)** and corresponding nucleus **(D)**. Negative control without anti-NMDA receptor antibody **(E)** and **(F)** DAPI counterstain; magnification 400x, scale bar 50 μm.

Evidence suggests that the glutamatergic system, especially the NMDA receptors, contribute to the pathophysiology of depression (Serafini et al., 2013). Thus, we decided to use differentiated mouse hippocampal neurons that express the NMDA receptor. After 48 h culture in differentiation medium as previously described by others (Suo et al., 2003), the immortalized mouse hippocampal neuronal HT-22 cells were transformed from an immature (Figure 1A) to a more mature neuronal state (Figure 1B). Undifferentiated mouse hippocampal neuronal cells had a more rounded morphology with few neurites or apparent synapses (Figure 1A). Upon exposure to differentiation conditions, the hippocampal cells changed to a neuron-like triangular shape and some of these cells were with extended neurites (Figure 1B). Besides morphological characteristics, using immunofluorescence-staining techniques, we also found functional indicators of neuronal differentiation, including NMDA receptor expression (Figures 1C–F).

### STW3-VI treatment of differentiated mouse hippocampal neuronal HT-22 cells stimulates neurite formation

**Figure 2:**
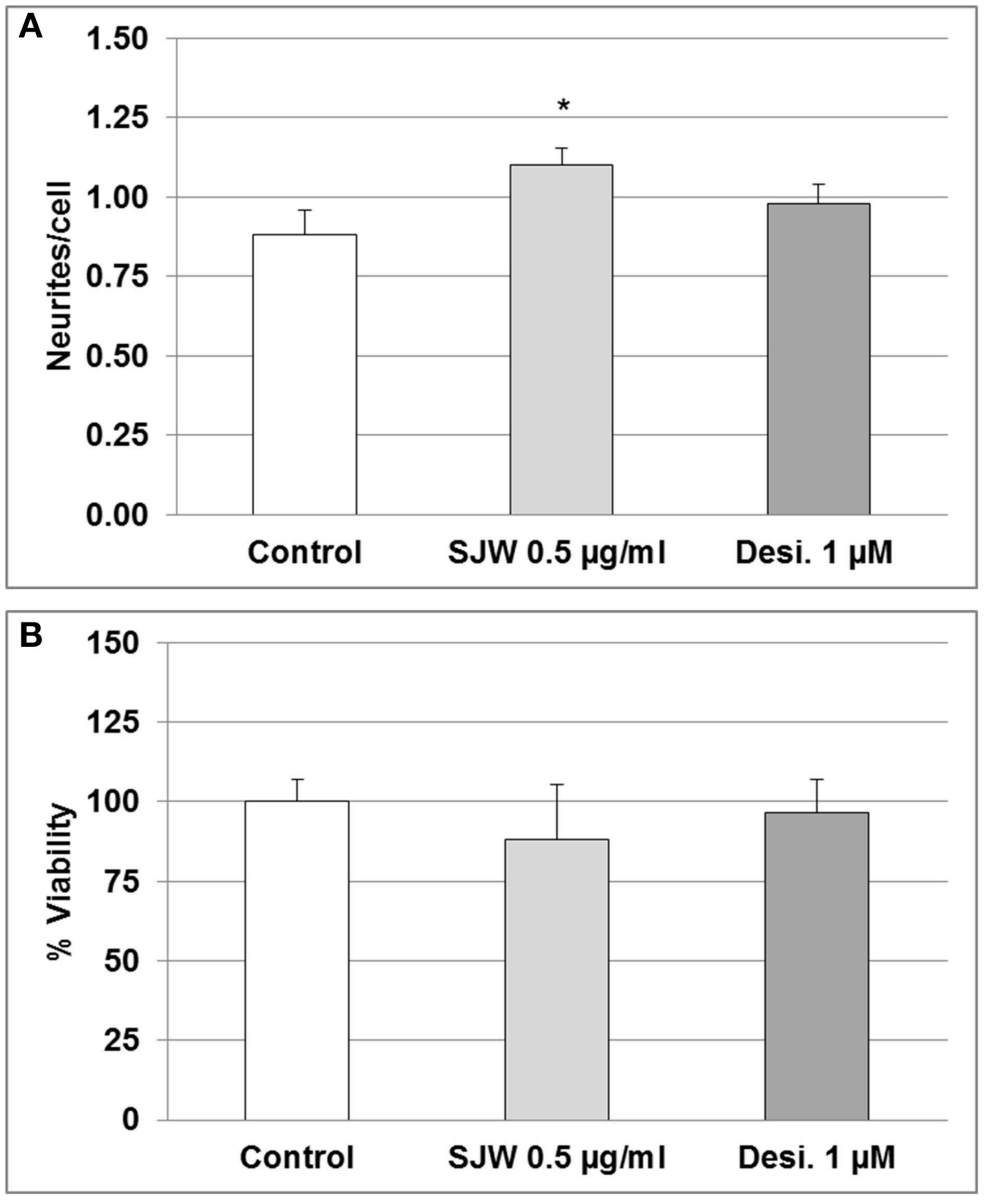
Neuronal plasticity and viability. **(A)** 0.5 μg/ml STW3-VI, St. John's wort extract (SJW) increased neurite outgrowth from differentiated mouse hippocampal HT-22 neurons after 48 h treatment, whereas 1.0 μM (~0.3 μg/ml) desipramine (Desi.) did not, in comparison with control (medium alone). **(B)** SJW and desipramine (Desi.) showed no significant effect on the viability of differentiated mouse hippocampal HT-22 neurons in comparison with control (medium alone). Quantification of neurites number/cell **(A)** or % viability **(B)** expressed as mean + SEM; TTEST, *p ≤ 0.05 vs. control; *n* = 8 independent experiments.

Several theories attempt to explain the causes of depression, including alterations in neurotrophic factors, changes of monoaminergic neurotransmission and dysregulation of adult hippocampal neurogenesis and plasticity (Wainwright and Galea, 2013). In this context, according to the concept of neural plasticity, the development of synaptic adaptations as well as dendritic spines are included (Wainwright and Galea, 2013). Therefore, we investigated the effect of STW3-VI on differentiated mouse hippocampal neuronal HT-22 cells *in vitro*. Incubation of differentiated mouse hippocampal HT-22 neurons with SJW (0.1, 0.5, or 1 μg/ml) or desipramine (0.1, 0.5, or 1 μM) did not significantly affect the viability (Figure S1). However, the neurite outgrowth was significantly increased (25%; *p* ≤ 0.05) after 48 h STW3-VI (0.5 μg/ml) treatment in comparison with the control (medium alone), whereas treatment with 1 mM desipramine (Desi.) showed no effect (Figure 2A). Cell viability was not significantly affected after treatment, neither with STW3-VI nor with desipramine (Figure 2B and Figure S1).

### STW3-VI protects differentiated mouse hippocampal neuronal HT-22 cells against glutamate and NMDA induced neurotoxicity

**Figure 3:**
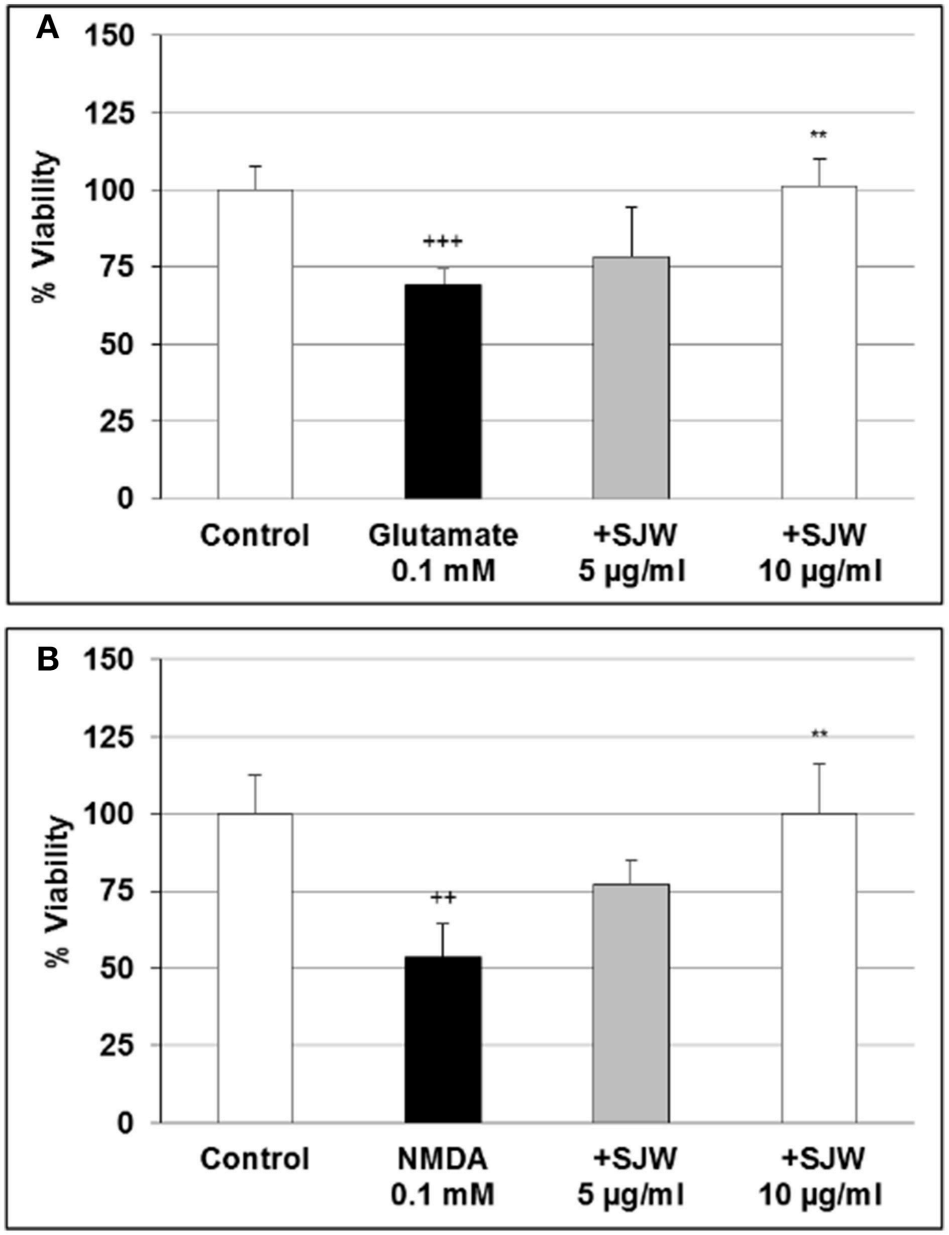
Effect of STW3-VI, St. John's wort extracts (SJW), against glutamate or NMDA induced cytotoxicity on differentiated mouse hippocampal HT-22 neurons. Protective effect of SJW pre-incubation (48 h) on glutamate **(A)** and NMDA **(B)** induced cytotoxicity. Glycin 5 mM was applied together with glutamate or NMDA. Data are presented as mean + SEM; TTEST, ^+++^*p* ≤ 0.001, ^++^*p* ≤ 0.01 significance vs. control; ^**^p ≤ 0.01 significance vs. glutamate or NMDA; *n* = 4–8 independent experiments.

Glutamate excitotoxicity has been shown to play a role in depression. Over-stimulation of NMDA receptors due to excessive glutamate concentrations result in excessive Ca^2+^ influx, which has been suggested to be implicated in the pathophysiology of many neurodegenerative diseases as well as in depression (Mody and MacDonald, 1995; Sattler and Tymianski, 2000). First we investigated the cytotoxic effects of various concentrations of glutamate and NMDA and found that 0.1 and 0.5 mM glutamate significantly (*p* < 0.01) inhibited the viability by 27.1 and 15.4% in comparison with the control (Figure S2A). NMDA significantly (*p* < 0.01) inhibited the viability by 39.0 and 26.2% after treatment with 0.1 and 0.5 mM, respectively (Figure S2B). To investigate the protective properties of STW3-VI in differentiated mouse hippocampal HT-22 neurons, we determined the viability after treatment with glutamate or NMDA as well as with 5 or 10 μg/ml or without STW3-VI pre-incubation (Figures 3A,B). Our data show that the cell viability was significantly decreased by 30.6% (*p* ≤ 0.001) and 46.2% (*p* ≤ 0.01) after treatment with 0.1 mM glutamate (Figure 3A) or 0.1 mM NMDA (Figure 3B), when compared to control. STW3-VI pre-treatment lead to a concentration-dependent abolishment (of glutamate/NMDA-induced decrease of viability) by 25% (*p* ≤ 0.01) and 50% (*p* ≤ 0.01) at 10 μg/ml STW3-VI in comparison to 0.1 mM glutamate (Figure 3A) or 0.1 mM NMDA (Figure 3B) treatment, indicating a cytoprotective effect of STW3-VI against glutamate- or NMDA-induced neurotoxicity.

### STW3-VI increases intracellular reduced glutathione in differentiated mouse hippocampal HT-22 neurons

**Figure 4:**
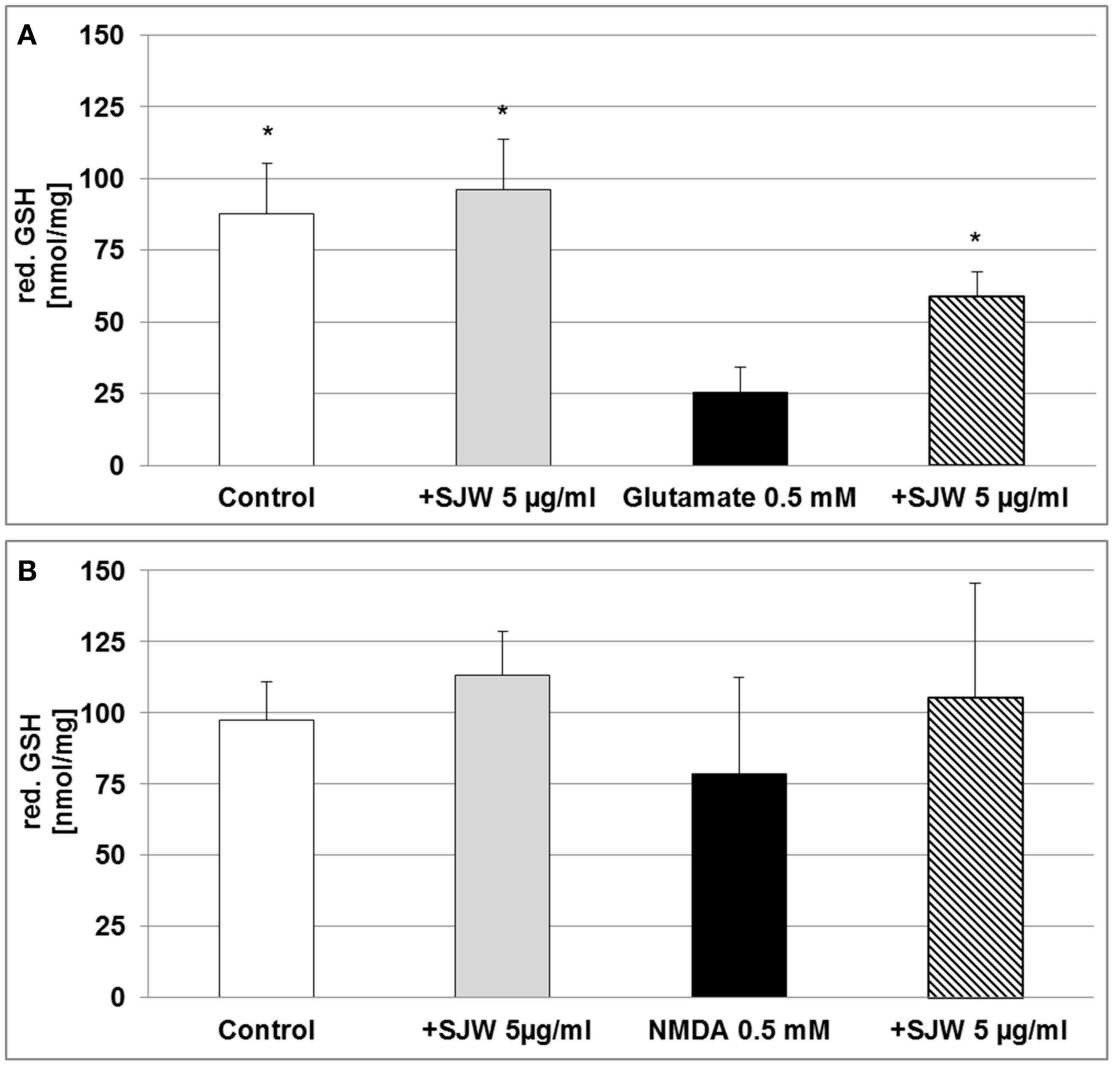
Effect of STW3-VI, St. John's wort extract (SJW), glutamate or NMDA on intracellular reduced glutathione (red. GSH) concentration of differentiated mouse hippocampal HT-22 neurons. **(A)** Glutamate (0.5 mM) decreased the intracellular red. GSH level. SJW pre-incubation prevented the glutamate-induced decrease of the intracellular red. GSH level. **(B)** NMDA treatment had no significant effect on the intracellular red. GSH status of differentiated mouse hippocampal HT-22 neurons. Data are presented as mean + SEM; TTEST, ^*^p ≤ 0.05, significance vs. glutamate; *n* = 4 independent experiments.

Cytoprotective enzymes and antioxidants are able to limit the cellular damage and thus, to control the toxicity of free radicals. The neurotransmitter glutamate can induce oxidative stress in cells of the brain, where a deficiency of reduced glutathione (GSH) has been observed and therefore, has been suggested to be of pathophysiological significance in depression (Gawryluk et al., 2010). Moreover, low GSH levels increase cellular sensitivity toward oxidative stress by accumulating reactive oxygen species (ROS). Treatment of differentiated mouse hippocampal HT-22 neurons with 0.5 mM glutamate significantly decreased intracellular GSH levels by 3.4-fold as compared to control (*p* < 0.05; Figure 4A), whereas pre-treatment with STW3-VI successfully prevented this GSH depletion. Thus, intracellular GSH was 2.3-fold increased in STW3-VI pretreated cells than in neurons incubated with glutamate alone (*p* < 0.05; Figure 4A). On the other hand, treatment with NMDA showed only a non-significant tendency to decrease intracellular GSH (Figure 4B), which was also prevented by STW3-VI. Thus, STW3-VI showed a protective effect against glutamate-induced oxidative stress by activation of the glutathione defense system (Figure 4A).

### STW3-VI inhibits the TNF release from LPS activated human THP-1 macrophages

**Figure 5:**
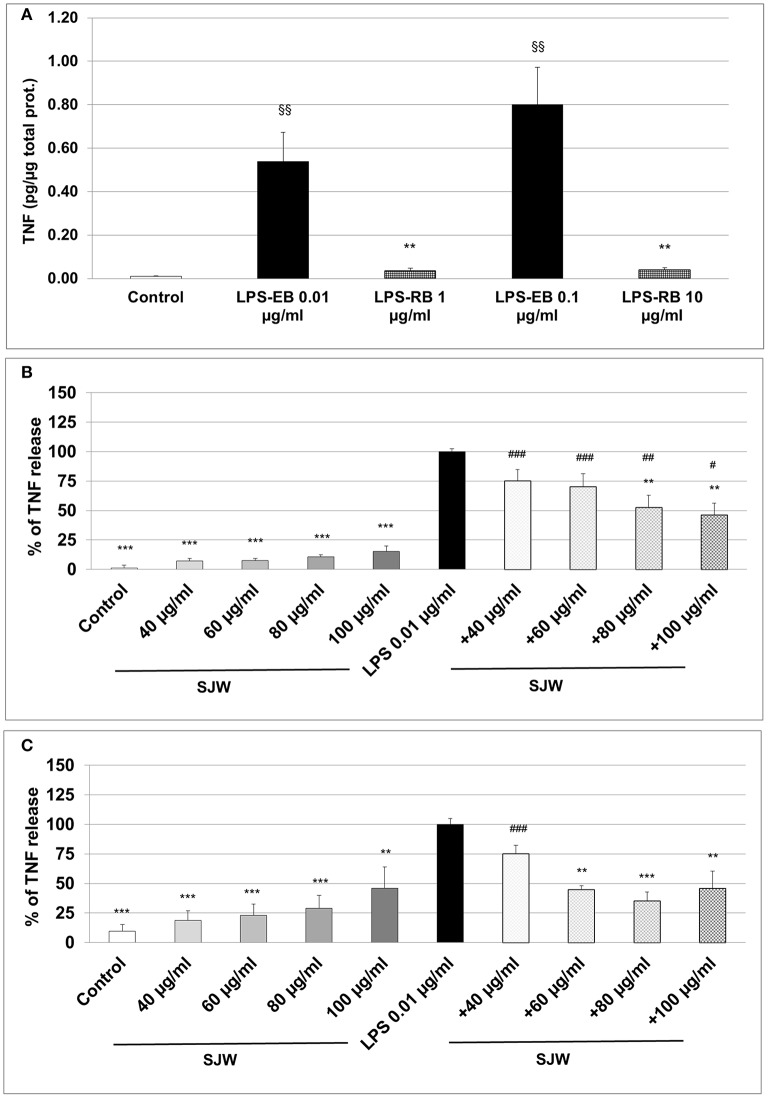
Tumor necrosis factor (TNF) alpha (quantification by ELISA). Effect of the pre-incubation of STW3-VI, St. John's wort extract (SJW) on the hTNF release of PMA differentiated human THP-1 macrophages. **(A)** LPS-EB (0.01 or 0.1 μg/ml) was co-incubated together with LPS-EB from *Rhodobacter sphaeroides* (LPS-RS) antagonist at 100-fold higher than the LPS-EB concentration and afterwards for 3 h with macrophages, as TLR4 binding assay specificity. TNF release after 24 h **(B)** or 48 h **(C)** SJW pre-treatment. SJW has shown anti-inflammatory effect against pro-inflammatory activation triggered by incubation with ultrapure LPS-EB, which is recognized only by TLR4. Data are presented as means + SEM; TTEST, §§*p* ≤ 0.01 vs. control **(A)**; ^**^p ≤ 0.01, ^***^p ≤ 0.001, significance vs. 3 h LPS treatment taken as 100% release or in pg. TNF /μg total protein **(A)**; ^#^*p* ≤ 0.05, ^##^*p* ≤ 0.01, ^###^*p* ≤ 0.001 vs. SJW without LPS; *n* = 4–7 independent experiments.

The measurement of the release of pro-inflammatory cytokines after treatment with a pro-inflammatory stimulus, such as LPS, is a method to analyse inflammatory processes *in vitro*; inhibition inhibition of this release by pharmacological intervention indicates anti-inflammatory properties of the substances properties. We used ultrapure-LPS-EB that is only recognized by TLR4, and activates pro-inflammatory signaling. LPS-EB (0.01 or 0.1 μg/ml) induced a significant 54-fold (*p* < 0.01) and 80-fold (*p* < 0.01) TNF release compared to the control (Figure 5A). Furthermore, as a control of the TLR4 specific binding, a competitive inhibition assay was performed with LPS of *R. sphaeroides* (LPS-RS). LPS-RS is a potent antagonist of LPS from pathogenic bacteria and binds with high affinity to the TLR4 but does not induce TLR4 signaling. Incubation of differentiated human THP-1 macrophages with the LPS-EB-antagonist—at 100-fold higher concentration than LPS-EB—abolished the hTNF release almost completely (Figure 5A). Additionally, incubation (24 or 48 h) of differentiated human THP-1 macrophages with various concentrations (20–100 μg/ml) of STW3-VI extracts showed no cytotoxic effect when compared with the control (Figure S3). Activation with 0.01 μg/ml LPS-EB induced a significant 90.9-fold (*p* < 0.001) or 10.3-fold (*p* < 0.001) TNF release, after 24 h (Figure 5B) or 48 h treatment (Figure 5C) when compared to the control (without LPS). Pre-incubation (24 h) of differentiated human THP-1 macrophages with STW3-VI (80 or 100 μg/ml) significantly (*p* ≤ 0.01) reduced the LPS-EB (0.01 μg/ml)-induced TNF release by 47.3 and 53.8% (Figure 5B). Prolonged (48 h) pre-incubation with STW3-VI (40–100 μg/ml) was even more effective in reducing LPS-EB-induced TNF release by 25.0–64.8% (Figure 5C). STW3-VI alone had no significant effect on the hTNF release in differentiated human THP-1 macrophages (Figures 5B,C). These results suggest an anti-inflammatory activity of STW3-VI through its inhibitory effect on the specific TLR4 signaling pathway.

### STW3-VI inhibits IL-6 and TNF-α mRNA gene expression in LPS-activated human THP-1 macrophages

**Figure 6:**
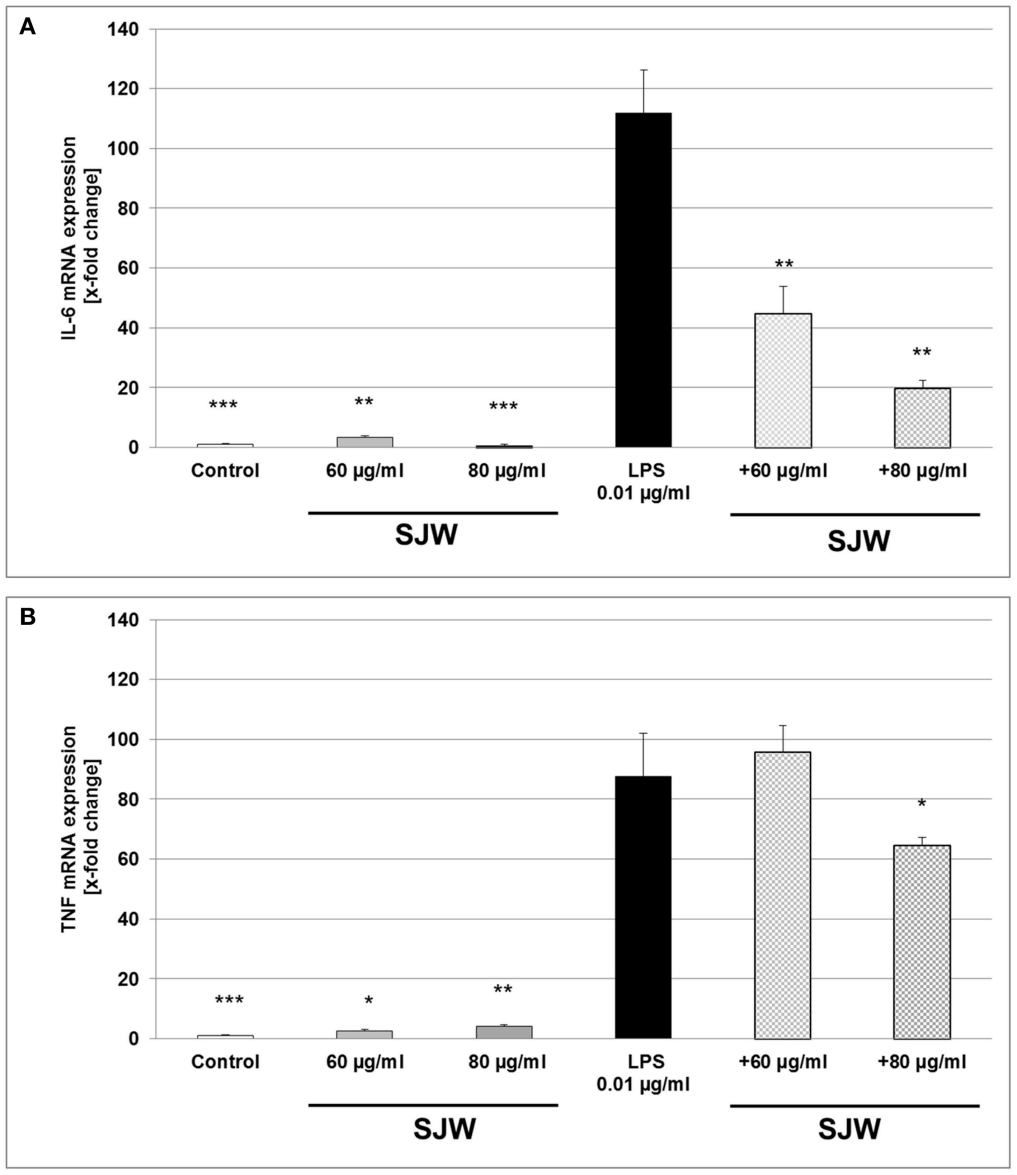
IL-6 and TNF mRNA gene expression analyses, using qRT-PCR. Effect of STW3-VI, St. John's wort extract (SJW) on the IL-6 **(A)** and TNF **(B)** mRNA expression of PMA differentiated human THP-1 macrophages after 24 h pre-treatment. SJW showed an anti-inflammatory effect on the IL-6 and TNF mRNA expression against the LPS pro-inflammatory activation. Data are presented as means + SEM; TTEST, ^*^p ≤ 0.05, ^**^p ≤ 0.01, ^***^p ≤ 0.001, significance vs. 3 h LPS treatment; *n* = 4 independent experiments.

To further explore the anti-inflammatory effect of STW3-VI on human THP-1 macrophages, we examined the expression of IL-6 and TNF mRNA after STW3-VI pre-treatment, followed by LPS-EB activation (3 h). There was a significant (*p* ≤ 0.001) 111.9-fold increase of IL-6 mRNA level after LPS treatment in comparison to the untreated control (medium alone; Figure 6A). However, pre-incubation with STW3-VI (60 or 80 μg/ml) significantly (*p* ≤ 0.01) attenuated the LPS-mediated IL-6 mRNA expression to 45- and 20-fold of control values (Figure 6A). TNF mRNA expression was also significantly increased by 87-fold (*p* ≤ 0.01) after stimulation with LPS compared to control (Figure 6B). Pre-incubation with 80 μg/ml, but not 60 μg/ml STW3-VI significantly (*p* ≤ 0.05) attenuated this increase to 63.9-fold (Figure 6B). Pre-treatment with STW3-VI alone had no significant influence on IL-6 or TNF mRNA expression (Figures 6A,B).

### STW3-VI inhibits IL-6 mRNA gene expression in differentiated hippocampal HT-22 neurons

**Figure 7:**
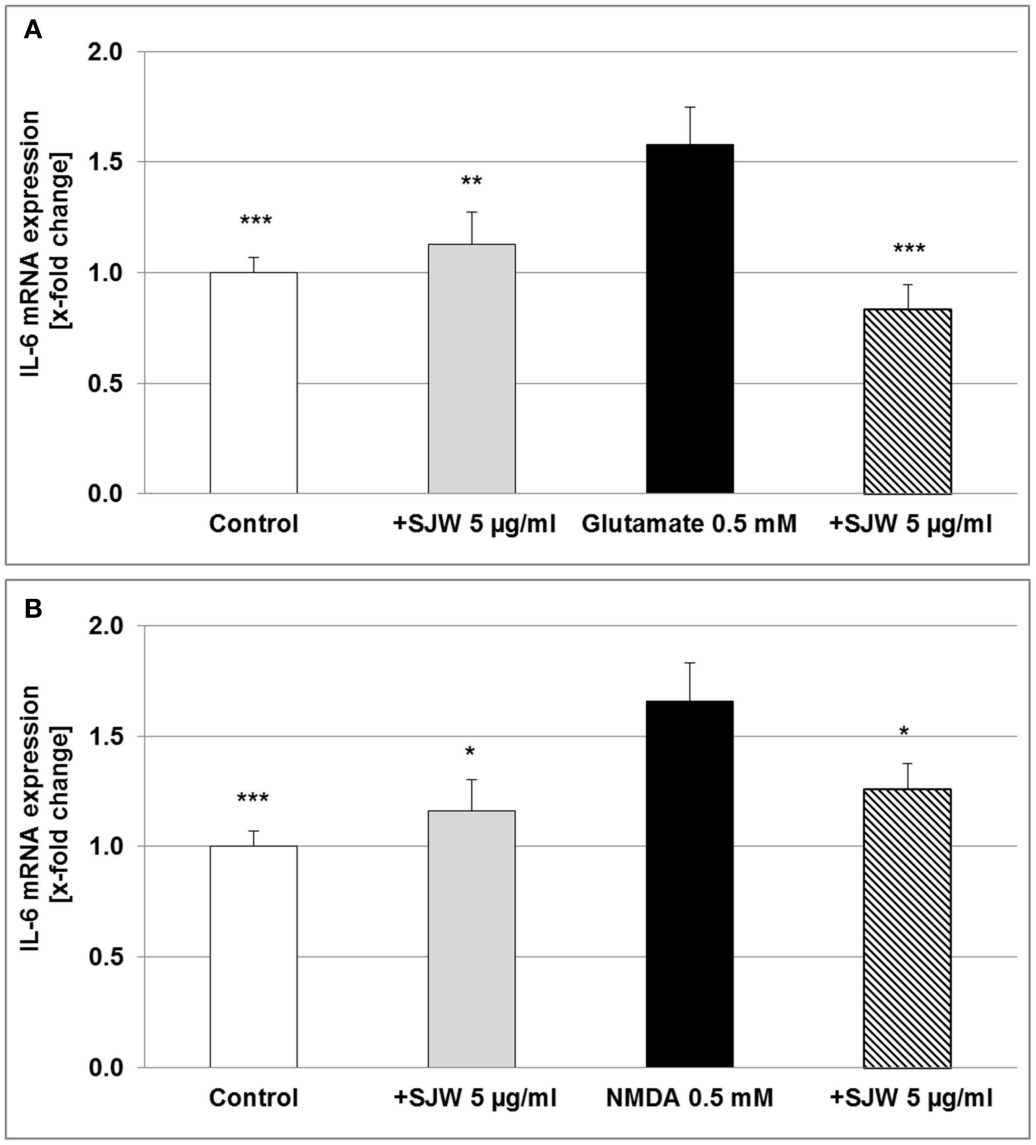
IL-6 mRNA gene expression analysis in differentiated mouse hippocampal HT-22 neurons, using qRT-PCR. The 24 h pre-incubation of differentiated mouse hippocampal HT-22 neurons with STW3-VI, St. John's wort extract (SJW) attenuated the glutamate **(A)** or NMDA **(B)** induced increase of IL-6 expression. Data are presented as mean + SEM; TTEST, ^*^p ≤ 0.05, ^**^p ≤ 0.01, ^***^p ≤ 0.001, significance vs. glutamate or NMDA treatment; *n* = 4 independent experiments.

Neurons, astrocytes, microglia and endothelial cells are the sources of IL-6 in the CNS, upon stimulation such as injury; abundant amounts of IL-6 are expressed and secreted (Erta et al., 2012). We found a significant increase of 58.0 and 66.0% (*p* ≤ 0.001) of IL-6 mRNA expression after treatment of differentiated mouse hippocampal HT-22 neurons with 0.5 mM glutamate or NMDA, compared to control (medium alone; Figure 7). Pre-treatment with 5 μg/ml STW3-VI significantly inhibited the pro-inflammatory effects of both, glutamate (*p* ≤ 0.001) or NMDA (*p* ≤ 0.05), even completely abolishing it for glutamate (Figure 7). These results also suggest a direct anti-inflammatory effect of STW3-VI on neurons.

## Discussion

STW3-VI (*Hypericum perforatum* L.) has been extensively used for the treatment of depression and anxiety disorders for its antidepressant and stress-reducing pharmacological properties (Butterweck et al., 1998; Butterweck, 2003; Linde et al., 2008; Grundmann et al., 2010) and contains hypericin, hyperforin and flavonoids (EMA/HMPC/101304/2008). So far, much research has been done in the identification and characterization of the active components of SJW with an antidepressant effect. The antidepressant properties may be a combination of the various components of SJW with potentially synergistic effect. In this context, hypericin and the flavonoids, or hyperforin display some effects in pharmacological models, but none of them alone appears indispensable for the therapeutical antidepressant efficacy (Butterweck and Schmidt, 2007). Until now there is not known a sole constituent of SJW extract with equivalent antidepressant activity which can represent the activity of the total extract. Thus, the whole extract must be considered as active with antidepressant properties.

Moreover, it was suggested that dysfunction of the glutamatergic system may be involved in the pathophysiology of depression (Skolnick et al., 1996), because depression was found to be associated with abnormal high accumulation of the neurotransmitter glutamate in the brain (Sun et al., 2010). Therefore, environmental stress, which increases glutamate, has dramatic impact on the morphology of the brain tissue and neuronal plasticity (Sun et al., 2010). Under pathological conditions, like brain injury and neurodegenerative diseases, glutamate may be, may be neurotoxic via excitotoxicity or oxidative stress (Lau and Tymianski, 2010; Okubo et al., 2010; Chhunchha et al., 2013). Because glutamate-induced excitotoxicity is mainly mediated by NMDA receptors (Lau and Tymianski, 2010) which are expressed by mature primary hippocampal neurons (Caldeira et al., 2007), immortalized cell lines, such as the immortalized mouse hippocampal neuronal HT-22 cell line are valuable tools for mechanistic studies. However, it is noteworthy to mention that mouse hippocampal HT-22 do not express NMDA receptors under normal culture conditions. Recent studies have shown that differentiated hippocampal HT-22 cells revealed more post-mitotic characteristics of neurons like outgrowth of neurites and expression of cholinergic receptors and markers (Suo et al., 2003; Liu et al., 2009; He et al., 2013). In accordance with these publications, upon differentiation conditions as described by others (Suo et al., 2003), we were able to obtain cells with neuronal characteristics. Our cells showed neuron-like triangular shape, extended neurites outgrowth and functioning NMDA receptor expression with capacity to respond to glutamate or NMDA neurotoxic stimulation. It is worth mentioning, that also undifferentiated HT-22 cells respond to glutamate-induced cytotoxicity, but only at concentrations of 2.5 mM (He et al., 2013) or 5 mM glutamate (Breyer et al., 2007), whereas in differentiated mouse hippocampal HT-22 neurons, cytotoxicity can be induced with glutamate concentrations as low as 0.03 mM (He et al., 2013). Under experimental conditions, using the protocol of Suo et al. (2003), in differentiated mouse hippocampal HT-22 neurons viability was reduced with 0.1 mM glutamate, additionally indicating that we used differentiated mouse hippocampal HT-22 neurons.

Recently, the association between depression, neuronal plasticity and glutamate/NMDA receptor–dependent synaptic potentiation as well as hippocampal activity has received much attention (Lüscher and Malenka, 2012; Wainwright and Galea, 2013). Neuronal plasticity is suggested as the ability of neurons and neural elements to adapt to intrinsic and extrinsic stimuli, e.g., chronic stress exposure which reduces the complexity and length of dendrites (Watanabe et al., 1992; London et al., 2013; Wainwright and Galea, 2013). The creation of new neurons is typically limited to certain brain regions such as the subgranular zone of the hippocampus (Kaplan and Hinds, 1977), and therefore, the use of natural products that promote neurogenesis and plasticity may be applicable to treat depressive stages. In this context, we found, that treatment of the differentiated mouse hippocampal HT-22 neurons with STW3-VI supported/enhanced the growth of neurites compared to neurons incubated with medium alone (control), suggesting that these neurotrophic properties might play a role in its positive effects on depression *in vivo*. Moreover, elevated concentration of extracellular glutamate is considered not only a main reason for the degeneration of neurons and/or cell death as observed in neurodegenerative diseases, but is also a risk factor for mood/anxiety disorders like depression (de Kloet et al., 2005; Grundmann et al., 2010; Hardingham and Bading, 2010). According to this concept, the neuroprotective properties of STW3-VI in glutamate-induced cell death in undifferentiated hippocampal HT-22 neurons have been previously described (Breyer et al., 2007). However, the resistance to excitotoxicity is due to lack of NMDA receptor expression in undifferentiated cells (He et al., 2013). Therefore, we used differentiated mouse hippocampal HT-22 neurons in the present study. Our results show that both, glutamate and NMDA are toxic for NMDA receptor positive, mouse hippocampal HT-22 neurons and, additionally, that STW3-VI protects against excitotoxicity induced by these compounds.

Elevated levels of glutamate in the brain can inhibit the synthesis of intracellular glutathione independently of the NMDA receptor activation status (Breyer et al., 2007) causing oxidative stress and cell death (Li et al., 1997). In line with these findings, our observations suggest that STW3-VI protects differentiated mouse hippocampal HT-22 neurons against the excitotoxic glutamate effect via the inhibition of the intracellular reduced GSH depletion, thus preventing glutamate-mediated oxidative stress-induced cytotoxicity. Furthermore, an earlier *in vivo* study using a chronic stress rat model of depression (Grundmann et al., 2010) showed that an administration of STW3-VI decreased the stress-induced TNF levels in plasma, providing novel evidence that the mechanism of STW3-VI action involves the link between immune-/neuroendocrine systems and oxidative stress defense. In accordance with these data, our study shows, that STW3-VI treatment leads to dose-dependent anti-inflammatory effects inhibiting TNF release from LPS-activated, PMA-differentiated human THP-1 macrophages as determined by ELISA. Our results reveal that STW3-VI is able to inhibit TNF and IL-6 mRNA expression, which is also in accordance with findings of others, who used different *in vitro* or *in vivo* experimental systems (Raso et al., 2002; Tedeschi et al., 2003; Huang et al., 2011; Abtahi Froushani et al., 2015) and thus, confirm the anti-inflammatory properties of STW3-VI in different experimental settings.

Furthermore, it has been reported that four constituents of *H. perforatum* L. extract, i.e., psuedohypericin, amentoflavone, quercetin, and chlorogenic, which acted synergistically, inhibited LPS-induced Prostaglandin E2 (PGE2), TNF and NO production in LPS-activated mouse RAW 264.7 macrophages, through SOCS3 (suppressor of cytokine signaling 3) activation, a part of the toll-like receptors (TLRs) pathway (Hammer et al., 2010; Huang et al., 2012). In our study, we have used an ultrapure LPS with high TLR4-affinity that only activates the TLR4 pathway for the pro-inflammatory activation of the human macrophages. Our results showed that STW3-VI inhibits activation of the pro-inflammatory signaling triggered via TLR4. The anti-inflammatory potential of the 4 compounds of STW3-VI, but not the whole extract, may be explained by the activation of the suppressor of cytokine signaling (SOCS) as described in RAW 264.7 macrophages (Huang et al., 2012). Moreover, LPS-induced neuroinflammation considerably decreases neurogenesis in rat brains (Ekdahl et al., 2003; Fujioka and Akema, 2010). In this context, the STW3-VI mediated anti-inflammatory effect in differentiated human THP-1 macrophages, as an *in vitro* model of systemic inflammation, can be recognized as potential inhibitor of neural inflammation and therefore, may encourage neurogenesis in patients with depression. Provided evidence reveal that pro-inflammatory cytokines play major regulatory roles in processes as neurogenesis, proliferation, neuronal migration and maturation (Schwartz and Shechter, 2010; Borsini et al., 2015). Finally, the concept of neuroinflammation being applicable to explain the development of neuropsychiatric disorders as depression, has most recently been supported by several publications (for review see Borsini et al., 2015; Mechawar and Savitz, 2016; Papageorgiou et al., 2016). In summary, we report that STW3-VI protects differentiated mouse hippocampal neurons against the glutamate or NMDA induced cytotoxicity *in vitro*. We describe an anti-inflammatory effect of STW3-VI on differentiated hippocampal neurons and a stimulation of neurites outgrowth, i.e., characteristic features of neuronal plasticity and possible neurogenesis, which indicates the importance of STW3-VI as potential anti-depressant therapeutical drug and further explains its multifaceted mechanism of action. Additionally, our data provide evidence that STW3-VI is an agent with anti-inflammatory effects on TLR4-LPS-stimulated differentiated human macrophages as *in vitro* model of systemic or resident macrophages.

## Author contributions

GB and RK were responsible for the conception and design of the study; and ASr, JH, and HS for the data collection, analysis, and image processing. GB wrote the manuscript; and ASz and RK revised it. HA-A and CK discussed the study concept and were responsible for the final approval of the version to be submitted. All authors read and approved the final manuscript.

### Conflict of interest statement

CK, HA-A are employed by Steigerwald Arzneimittelwerk GmbH. All the other authors declare that the research was conducted in the absence of any commercial or financial relationships that could be construed as a potential conflict of interest.
