# Supplementary material for: Neurotrophic, Cytoprotective, and Anti-inflammatory Effects of St. John's Wort Extract on Differentiated Mouse Hippocampal HT-22 Neurons
Source: Front Pharmacol. 2018 Jan 18;8:955. doi: 10.3389/fphar.2017.00955 (PMC5778116; doi:10.3389/fphar.2017.00955)
Supplement: Supplementary file 1 [file Image1.PDF]

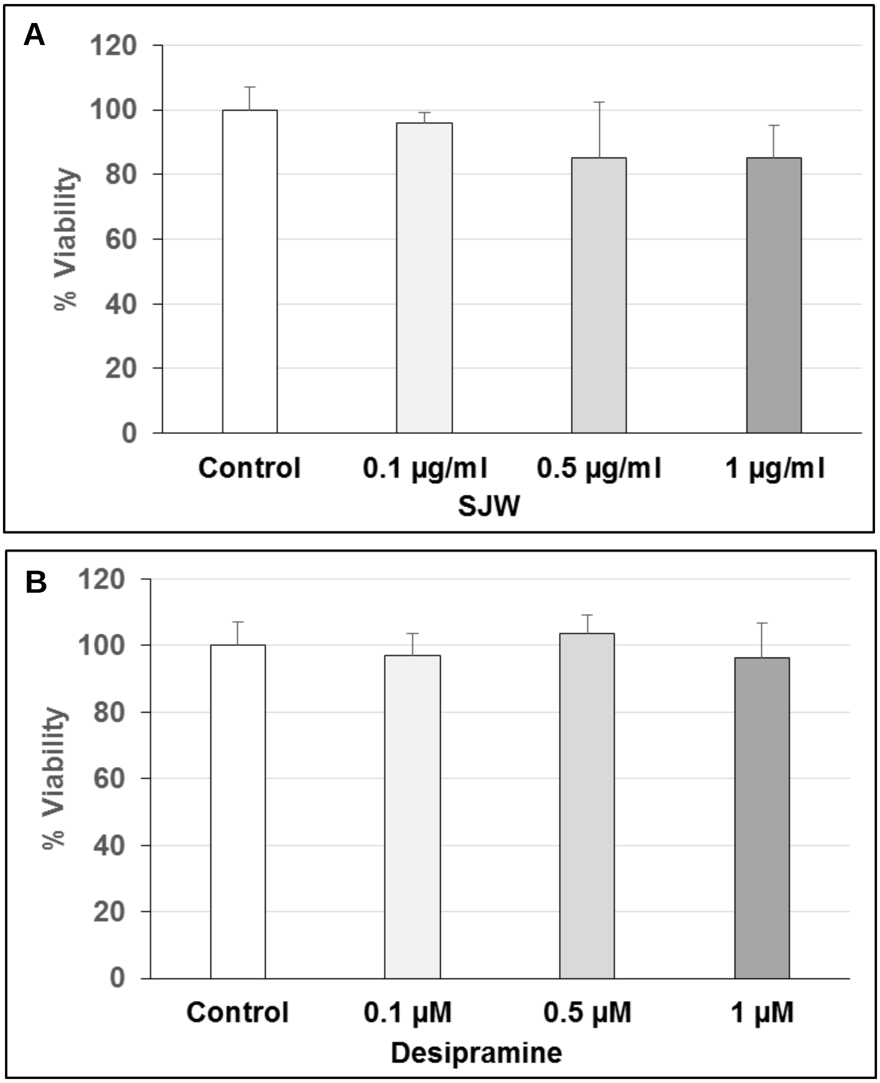

Supplementary Fig. S1. Neuronal viability. Effect of various concentrations of A) SJW and B) desipramine (Desi.) on the viability of differentiated mouse hippocampal HT-22 neurons in comparison with control (medium alone) after 48 h treatment. Data are shown as mean + SEM; TTEST, vs. control; n=8 independent experiments.
