# Supplementary material for: Neurotrophic, Cytoprotective, and Anti-inflammatory Effects of St. John's Wort Extract on Differentiated Mouse Hippocampal HT-22 Neurons
Source: Front Pharmacol. 2018 Jan 18;8:955. doi: 10.3389/fphar.2017.00955 (PMC5778116; doi:10.3389/fphar.2017.00955)
Supplement: Supplementary file 2 [file Image2.PDF]

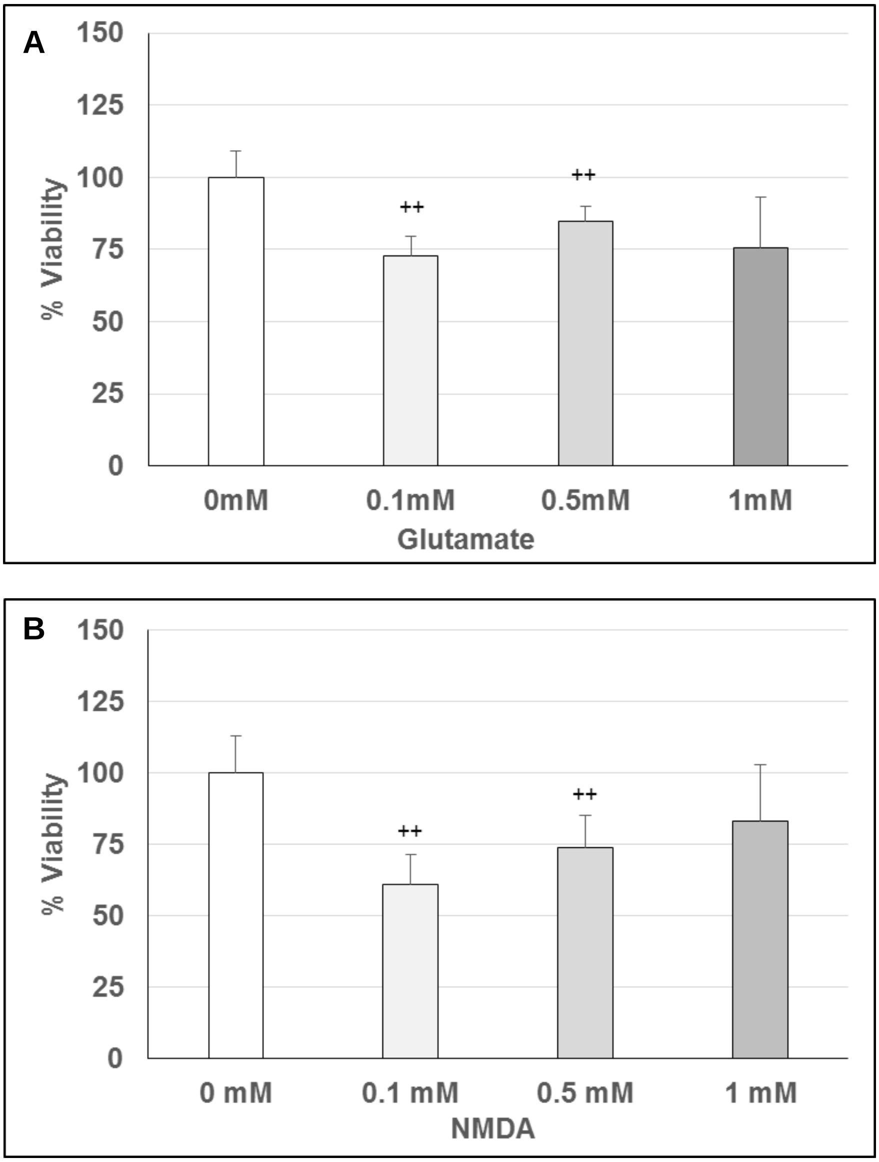

Supplementary fig. S2. Glutamate or NMDA induced cytotoxicity on differentiated mouse hippocampal HT-22 neurons. Quantification of the viability after 6 h glutamate (A) or NMDA treatment (B). Glycin 5 mM was applied together with glutamate or NMDA. Data are presented as mean + SEM; TTEST, $^{++}p \leq 0.01$ significance vs. control; n=4-8 independent experiments.
