# Supplementary material for: Neurotrophic, Cytoprotective, and Anti-inflammatory Effects of St. John's Wort Extract on Differentiated Mouse Hippocampal HT-22 Neurons
Source: Front Pharmacol. 2018 Jan 18;8:955. doi: 10.3389/fphar.2017.00955 (PMC5778116; doi:10.3389/fphar.2017.00955)
Supplement: Supplementary file 3 [file Image3.PDF]

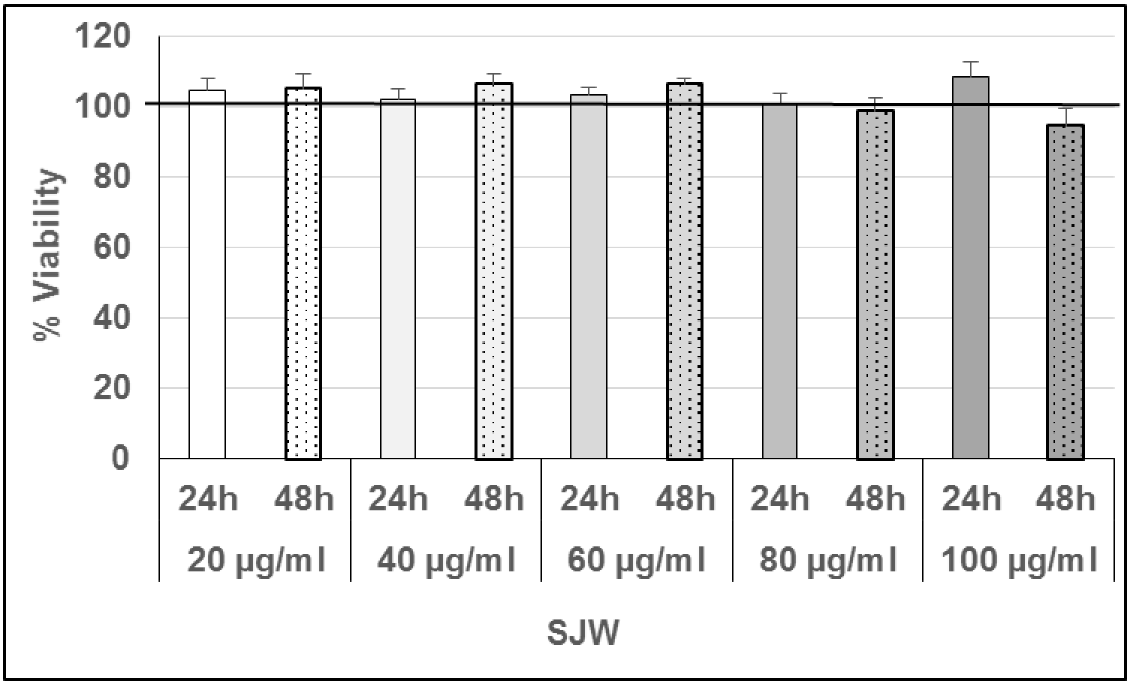

Supplementary Fig. S3. Viability of differentiated human THP-1 macrophages after incubation (24h or 48h) with various concentrations (20 - 100 µg/ml) of STW3-VI extracts. Data are presented as means + SEM; TTEST vs. control (100 % viability), n=4-7 independent experiments.
